# Srsf10 and the minor spliceosome control tissue-specific and dynamic SR protein expression

**DOI:** 10.7554/eLife.56075

**Published:** 2020-04-27

**Authors:** Stefan Meinke, Gesine Goldammer, A Ioana Weber, Victor Tarabykin, Alexander Neumann, Marco Preussner, Florian Heyd

**Affiliations:** 1 Freie Universität Berlin, Institute of Chemistry and Biochemistry, Laboratory of RNA BiochemistryBerlinGermany; 2 Institute of Cell Biology and Neurobiology, Charité-Universitätsmedizin Berlin, corporate member of Freie Universität Berlin, Humboldt-Universität zu Berlin, and Berlin Institute of HealthBerlinGermany; Case Western Reserve UniversityUnited States; Columbia UniversityUnited States

**Keywords:** minor spliceosome, SR proteins, alternative splicing, SRSF10, Human, Mouse

## Abstract

Minor and major spliceosomes control splicing of distinct intron types and are thought to act largely independent of one another. SR proteins are essential splicing regulators mostly connected to the major spliceosome. Here, we show that *Srsf10* expression is controlled through an autoregulated minor intron, tightly correlating Srsf10 with minor spliceosome abundance across different tissues and differentiation stages in mammals. Surprisingly, all other SR proteins also correlate with the minor spliceosome and *Srsf10*, and abolishing *Srsf10* autoregulation by Crispr/Cas9-mediated deletion of the autoregulatory exon induces expression of all SR proteins in a human cell line. Our data thus reveal extensive crosstalk and a global impact of the minor spliceosome on major intron splicing.

## Introduction

Alternative splicing (AS) is a major mechanism that controls gene expression (GE) and expands the proteome diversity generated from a limited number of primary transcripts (Nilsen and Graveley, 2010). Splicing is carried out by a multi-megadalton molecular machinery called the spliceosome of which two distinct complexes exist. The more abundant major spliceosome that consists of the U1, U2, U4, U5, U6 small nuclear ribonucleoprotein particles (snRNPs) and multiple non-snRNP splicing factors. Additionally, the cells of most eukaryotes contain the minor spliceosome, which is composed of the minor-specific snRNPs U11, U12, U4atac, U6atac, and the shared U5 snRNP. While the major spliceosome catalyzes splicing of around 99.5% of all introns, mainly so-called U2-type introns with the characteristic GT-AG splice sites, the minor spliceosome recognizes introns of the U12-type containing non-consensus AT-AC splice sites and distinct branch point and polypyrimidine sequences (Jackson, 1991; Turunen et al., 2013). However, despite its low abundance, the minor spliceosome plays a fundamental role in ontogenesis, as deficiencies in minor spliceosome activity or minor intron splicing are lethal or result in developmental defects and disorders (Doggett et al., 2018; Verma et al., 2018).

AS is highly regulated by *cis*-acting splicing enhancer and silencer elements, which are recognized by various RNA binding proteins, such as SR proteins and heterogeneous nuclear ribonucleoproteins (Hastings et al., 2001). The protein family of SR proteins, with 13 canonical members in humans, is characterized by an arginine and serine rich domain (RS domain) (Manley and Krainer, 2010). Aside from their role in mRNA nuclear export and GE they are essential regulators of AS (Long and Caceres, 2009). Every SR protein contains ultraconserved elements in alternative exons that control the presence of premature translation termination codons (PTC). This allows them to regulate their own abundance through nonsense-mediated decay (NMD) (Lareau et al., 2007). While many SR proteins and RBPs use autoregulation to maintain a stable expression level (Müller-McNicoll et al., 2019), their expression level changes in a tissue-specific manner (Wang et al., 2008; Olthof et al., 2019). Therefore, mechanisms aside from autoregulation are most likely employed to control SR protein levels under different conditions, for instance in different tissues or during differentiation. However, the mechanisms that coordinately regulate SR protein expression levels remain elusive.

SRSF10 is a unique SR protein, as it activates splicing in its phosphorylated state but becomes a general splicing inhibitor upon dephosphorylation (Feng et al., 2008; Zhou et al., 2014). We used SRSF10 as a case study of how tissue-specific differences in SR protein levels can be achieved by employing an autoregulatory feedback loop. We show that Srsf10 recognizes a highly conserved splicing enhancer element within its own pre-mRNA, which results in the production of a non-protein coding mRNA isoform and thereby the regulation of its own expression level. An additional layer of *Srsf10* regulation is added by the presence of competing major and minor splice sites which control this autoregulatory AS event. The minor splice site leads to the formation of the protein-coding mRNA, whereas splicing mediated by the major spliceosome leads to the non-protein-coding mRNA. Consequently, *Srsf10* levels correlate with the level of the minor spliceosome in a tissue- and developmental stage-specific manner. Surprisingly, we also found that the expression levels of most other SR proteins correlate with *Srsf10* expression. This is directly mediated through the levels of *Srsf10* and the competition between major and minor splice sites, as CRISPR/Cas9-mediated removal of the autoregulatory exon 3 of *Srsf10* increases not only the expression of *Srsf10*, but also the expression of the other SR proteins. These data connect the minor spliceosome with *Srsf10* and SR protein expression in a tissue- and differentiation state-specific manner. We thus reveal a mechanism that coordinately controls SR protein expression in different cellular conditions and that connects the minor spliceosome with global (alternative) splicing of major introns.

## Results and discussion

### Srsf10 autoregulates its own splicing and expression

**Figure 1.**
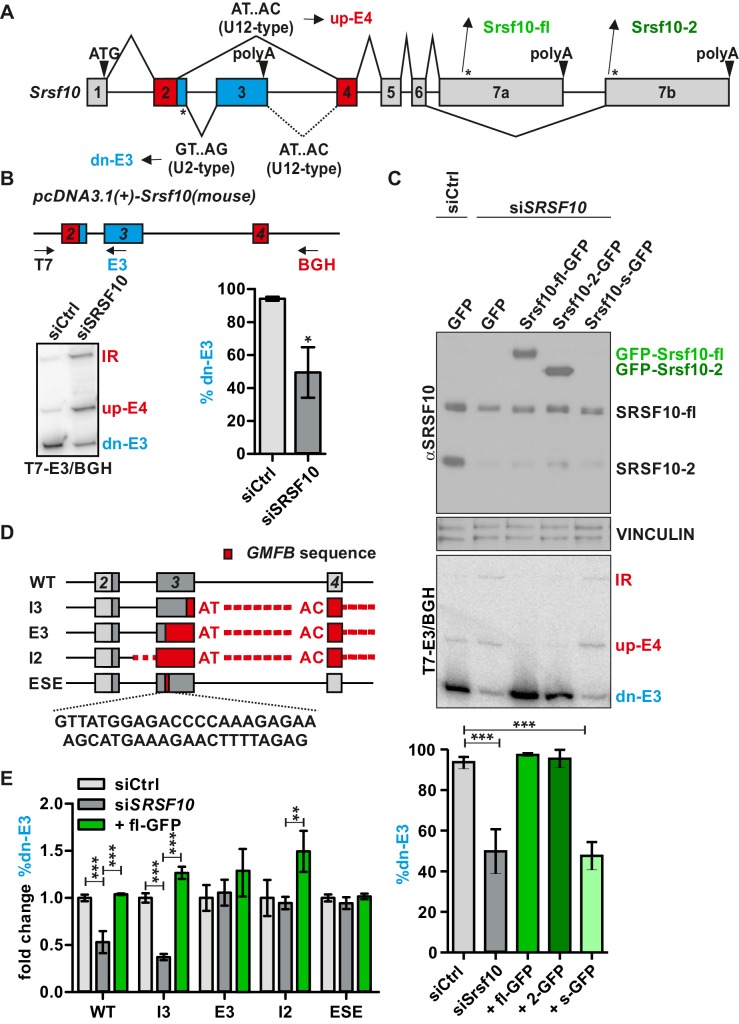
Srsf10 autoregulates its own splicing through a conserved enhancer in exon 3. (**A**) Schematic of the exon/intron structure of Srsf10. Usage of the downstream (dn) major splice site in Srsf10 exon 2 (GT.AG, U2-type) leads to exon 3 inclusion and a non-protein coding isoform, while usage of the upstream minor splice site (AT.AC, U12-type) results in exon 4 inclusion. A minor 5’ splice site in exon 3 is present but not used in the endogenous context (dotted lines). * indicate stop codons. (**B**) Srsf10 minigene splicing upon siRNA-mediated knockdown of endogenous Srsf10. Top: exon/intron structure of the Srsf10 minigene reporter containing mouse exons 2 to 4 (and complete intervening introns) with indicated primer binding sites (arrows). HeLa cells were transfected with control siRNA (siCtrl) or against human Srsf10 (siSrsf10), incubated for 24 hr followed by minigene transfection. After 48 hr splicing was analyzed with the indicated primers. Bottom: exemplary gel and quantification of the dn-E3 isoform (n = 5, mean ± SD). (**C**) Knockdown and rescue of SRSF10. Top: Western Blot of SRSF10 after siRNA-mediated knockdown and transfection with overexpression vectors for the different GFP-tagged Srsf10 isoforms. VINCULIN was used as loading control. Middle: Exemplary gel of Srsf10 minigene splicing upon knockdown and rescue. Bottom: Quantification of the dn-E3 isoform (n ≥ 3, mean ± SD). (**D**) Exon/intron structure of the Srsf10 minigenes used for mutational analysis. Exon and intron sequences were replaced by sequences containing a minor intron from glia maturation factor beta (Gmfb exons 4 to 5 including the minor intron 4, marked in red). Below the sequence of the identified ESE is shown. (**E**) Quantification of Srsf10 minigene splicing upon knockdown and rescue. HeLa cells were transfected with the mutated minigenes (**D**) and analyzed as in (**B**). Splicing of mutants is shown relative to the wt from (**B**) and for each mutant relative to the Ctrl siRNA (n = 5, mean ± SD). Student’s t test-derived p values *p<0.05, **p<0.01, ***p<0.001.

Autoregulation has been described for many RBPs including most SR proteins (Lejeune et al., 2001; Sureau, 2001), but not for Srsf10. *Srsf10* represents a particularly interesting example as its conserved region contains two competing 5’ splice sites in exon 2 (E2), which are recognized by either the minor or the major spliceosome. The upstream (up) minor splice site is coupled to E4 inclusion and production of a protein coding mRNA, while use of the downstream (dn) major splice site is coupled to E3 inclusion, the presence of a PTC and the use of an alternative polyadenylation site in E3 (Figure 1A). The dn-E3 variant is not a canonical NMD target, as the stop codon in E2 is less than 50 nucleotides upstream of the E2/3 junction (Nagy and Maquat, 1998) and could thus encode for a hypothetical short protein (Srsf10-s, see below). To investigate whether AS of the competing minor and major splice site in exon 2 of *Srsf10* depends on an autoregulatory feedback loop, we generated an *Srsf10* minigene containing mouse exons 2 to 4 (Figure 1B, top). We transfected this minigene into human HeLa cells and investigated AS after knocking down the endogenous *SRSF10*. These experiments revealed strong autoregulation, as *SRSF10* knockdown decreased the dn-E3 isoform and increased the up-E4 product and retention of intron 2 (IR, Figure 1B, bottom). We confirmed the knockdown of *SRSF10* (Figure 1—figure supplement 1A and B) and observed a reduced E3/E4 ratio for endogenous *SRSF10* mRNA (Figure 1—figure supplement 1B), consistent with SRSF10 activating E3 inclusion.

**Figure 1—figure supplement 1.**
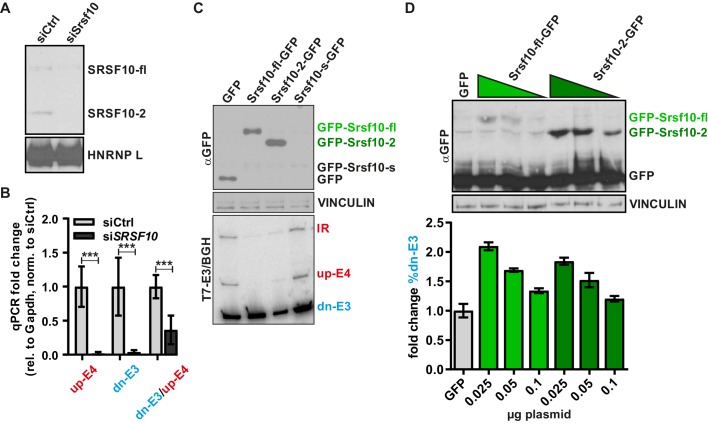
Srsf10 autoregulates its own splicing. (**A, B**) Confirmation of the Srsf10 knockdown. In (**A**) SRSF10 protein levels were investigated by Western Blot using an SRSF10-specific antibody. Note that both SRSF10 variants (fl and −2) are strongly reduced. The remaining signal for SRSF10-fl could indicate some unspecific detection of a different (SR)-protein. HNRNP L served as a loading control. In (**B**) confirmation of the Srsf10 knockdown on mRNA level is shown. The expression of the different Srsf10 isoforms is shown relative to Gapdh and control siRNA (n ≥ 6, mean ± SD). Note the reduced dn-E3/up-E4 ratio after knockdown of Srsf10. (**C**) Overexpression of SRSF10 variants as in Figure 1C. Top: Overexpression of the SRSF10 variants was investigated with an antibody detecting the GFP epitope. SRSF10-s was not detectably expressed. VINCULIN served as a loading control. Bottom: Representative image of splicing analysis of co-transfected minigenes. (**D**) Dose-dependent Srsf10 autoregulation. HeLa cells were cotransfected with the minigene, decreasing amounts of SRSF10-fl or −2 (0.1, 0.05, or 0.025 µg plasmid) and adjusted amounts of GFP. Expression was confirmed using an antibody against GFP (top). Note the higher expression levels of SRSF10-2. VINCULIN served as a loading control. Bottom: Splicing analysis of cotransfected minigenes (n = 2, mean ± SD). Note that barely detectable amounts of SRSF10 already change splicing with a stronger effect of SRSF10-fl, especially prominent given the lower expression level of this variant.

**Figure 1—figure supplement 2.**
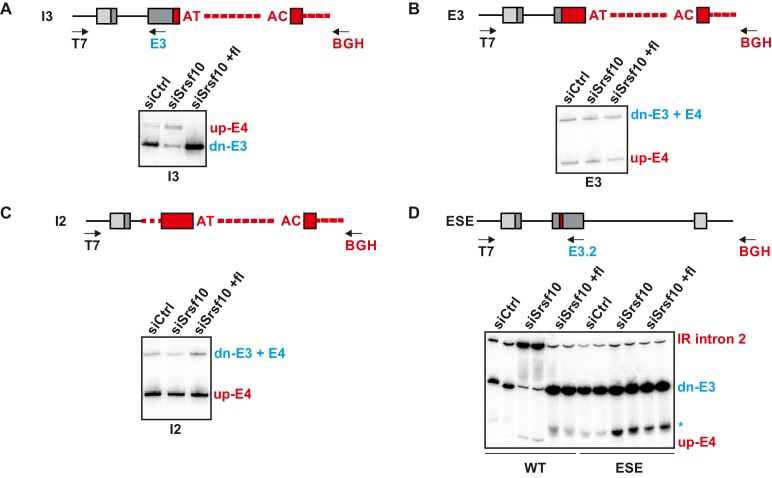
Srsf10 autoregulates its own splicing through a conserved enhancer in exon 3. (**A–D**) Analysis of mutated minigene splicing via radioactive RT-PCR in knockdown (siSrsf10) and knockdown-rescue with SRSF10-fl (siSrsf10 +fl) conditions. In each panel on top the investigated minigene and the primers used are shown. Below, a representative gel with indicated splicing isoforms. The I3 mutant (**A**) was investigated as the WT in Figure 1C. After mutation of the polyA site in exon 3, mutants E3 and I2 (**B and C**) could be investigated with only BGH reverse, amplifying both splicing isoforms. The ESE mutant (**D**) was investigated with a second reverse primer in exon 3 (E3.2), and is shown in comparison to a transfected WT minigene in duplicates. The asterisk marks a degradation product most likely of dn-E3. Note that SRSF10 dependent generation of up-E4 and IR is abolished in the mutant. We observe less IR in the mutant in control condiitons, indicating that the SRSF10-dependent splicing enhancer sequence has an additional role in controlling splicing.

**Figure 1—figure supplement 3.**
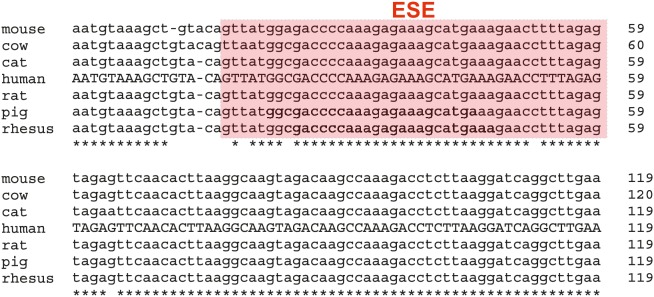
Alignment of the 5’ sequence of Srsf10 exon 3, which includes the identified ESE region (marked in red), showing high conservation across 7 mammals.

AS of *Srsf10* results in three possible protein isoforms: SRSF10-fl (inclusion of exon 7a), SRSF10-2 (inclusion of exon 7b, see Figure 1A), and a hypothetical protein resulting from exon 3 inclusion (stop codon within the dn-E2 sequence, see Figure 1A). To investigate the activity of these protein isoforms in regulating AS, we performed rescue experiments with GFP-tagged Srsf10 mouse variants (not targeted by the human-specific siRNA). A Western blot against Srsf10 shows expression of GFP-Srsf10-fl and GFP-Srsf10-2 close to endogenous levels, while GFP-Srsf10-s was not detectable (Figure 1—figure supplement 1C, top). Consistently, Srsf10-fl and Srsf10-2 clearly rescue exon 3 mis-splicing caused by the knockdown of endogenous *SRSF10*, while transfecting Srsf10-s has no effect on *Srsf10* AS (Figure 1C). We obtained the same result when overexpressing the different Srsf10 variants with no knockdown of the endogenous protein, confirming Srsf10-fl and Srsf10-2 as activators of exon 3 inclusion and Srsf10-s as a barely expressed protein (Figure 1—figure supplement 1C). Since the presence of Srsf10-s is hardly detectable, even with a stabilizing GFP tag, we assume that this protein variant is highly instable and does not have a biological function. To compare the activities of Srsf10-fl and Srsf10-2, we performed titration experiments. This demonstrated highly sensitive *Srsf10* autoregulation, with Srsf10-fl, despite its lower expression levels, being a more potent activator of E3 inclusion than Srsf10-2 (Figure 1—figure supplement 1D). These data identify Srsf10 as an activator of the major intron between exons 2 and 3 and suggest that Srsf10 autoregulates its own expression level via a negative feedback loop, as higher Srsf10 levels result in the formation of the non-protein-coding dn-E3 isoform.

To identify the cis-regulatory element required for autoregulation, we used systematic mutational analysis of the *Srsf10* minigene (Figure 1D, Figure 1—figure supplement 2, A–D). First, we replaced sequences downstream of exon 3 by human Gmfb sequences from exons 4 to 5, including a minor intron (mutant I3, see ‘Material and Methods’ for details). This reflects the endogenous situation, as *Srsf10* exon 3 contains a minor 5’ splice site, which, however, is rarely used, since the polyadenylation site in exon 3 appears to be dominant (Figure 1A). The resulting minigene clearly remains responsive to *SRSF10* knockdown and overexpression (Figure 1E, I3). In contrast, replacing sequences starting from exon 3 (E3) or in intron 2 (I2) by *Gmfb* sequences results in splicing unresponsive to *SRSF10* knockdown and barely responsive to SRSF10 overexpression (Figure 1E; mutants E3 and I2). These data suggest that SRSF10 controls its own splicing via binding to exon 3, which, indeed, contains a GA-rich element representing the previously identified SRSF10 consensus binding site (Shin and Manley, 2002; Zhou et al., 2014). Replacing nucleotides 17 to 60 of exon 3 by *GMFB* exon ﻿4 sequence was sufficient to abolish SRSF10-mediated AS (Figure 1D and E, ESE; Figure 1—figure supplement 3), thus identifying this GA-rich element as an SRSF10-dependent exonic splicing enhancer (ESE). Together, these data identify a highly conserved element in *Srsf10* exon 3 which is necessary for an autoregulatory feedback loop that controls Srsf10 expression levels.

### The minor spliceosome controls Srsf10 expression

**Figure 2.**
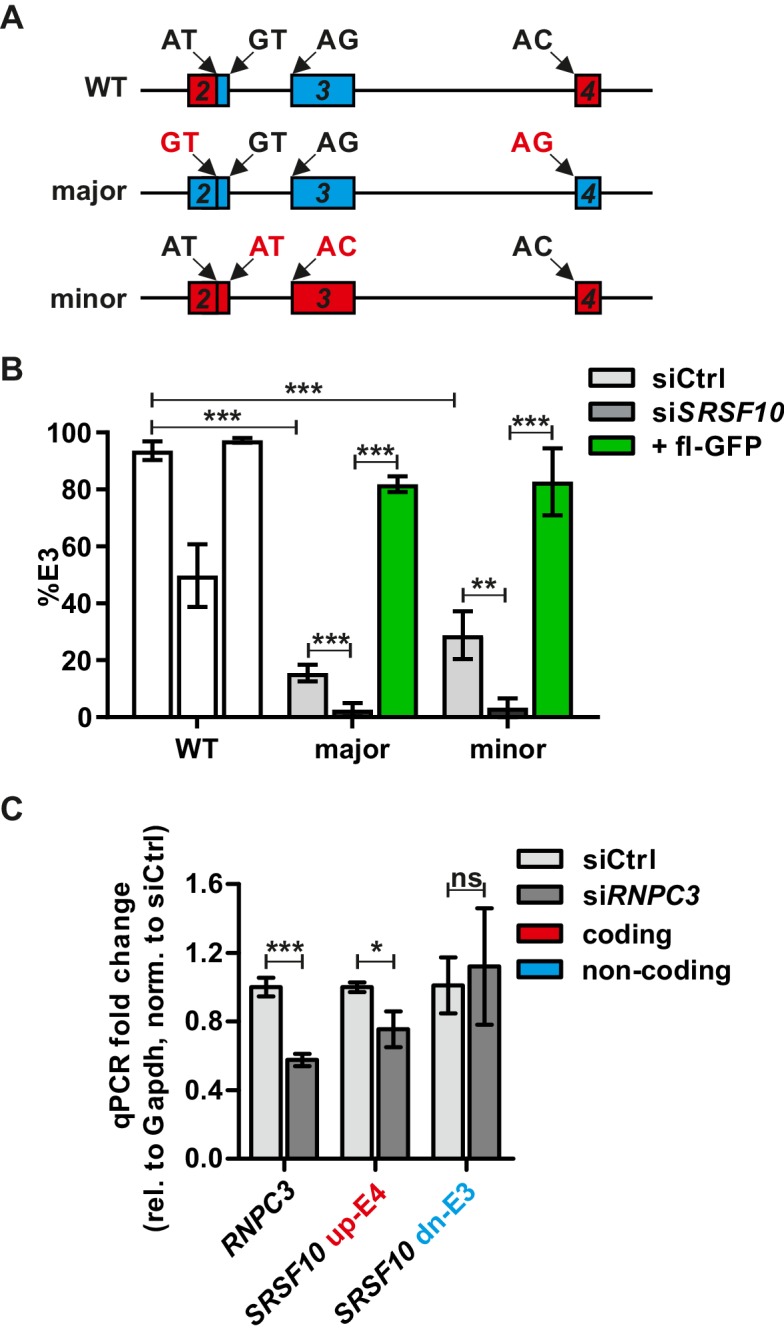
The minor spliceosome controls Srsf10 expression. (**A**) Schematic of the exon/intron structure of the Srsf10 WT and mutated minigenes harboring only major (GT.AG, blue) or minor (AT.AC, red) splice sites. Mutated splice sites are marked in red. (**B**) Minigenes from (**A**) were analyzed as in Figure 1. Quantification of splicing-sensitive RT-PCRs is shown relative to the WT from Figure 1B (n = 4, mean +/- SD). A representative gel is shown in Figure 2—figure supplement 1. (**C**) RT-qPCRs confirm siRNA-mediated knockdown of Rnpc3 (left) and changes in Srsf10 expression levels in HEK293. Expression relative to Gapdh and normalized to siCtrl (n = 3, mean +/- SD). Student’s t test-derived p values *p<0.05, **p<0.01, ***p<0.001.

Exon 2 of *Srsf10* contains two competing 5’ splice sites, which are specifically recognized by either the minor or the major spliceosome. To investigate the relevance of these splice sites for *Srsf10* autoregulation, we generated mutated minigenes containing either only major or only minor splice site (Figure 2A) and analyzed AS of the resulting minigenes. Mutated minigenes remained clearly responsive to SRSF10 knockdown and rescue, demonstrating that SRSF10 can regulate AS through both major and minor spliceosomes. However, in the control conditions (siCtrl), we observed that both minor-only or major-only minigenes show a strong increase in the use of exon 4 (Figure 2B). Exon 3 is hardly included at all in any of the two minigenes. Additionally, in the presence of a directly competing upstream splice site in exon 2, the downstream splice site is no longer used (Figure 2B, Figure 2—figure supplement 1). These data indicate that, in vivo, the use of the minor splice site that leads to productive *Srsf10* splicing, is reduced through the presence of a competing major splice site. This arrangement could render *Srsf10* expression susceptible to dynamic control through alterations in the activity of the minor spliceosome. To directly investigate this hypothesis, we inhibited minor spliceosome activity by performing an siRNA-mediated knockdown of the essential U11/U12 snRNP component *RNPC3* (Figure 2C). Indeed, the expression of the coding *SRSF10* mRNA (up-E4) was significantly decreased, while the levels of the non-coding dn-E3 mRNA are unaffected or slightly increased (Figure 2C). This result indicates that the abundance of the minor spliceosome directly correlates with *Srsf10* GE through controlling productive vs. non-productive AS. Furthermore, regulation through the activity of the minor spliceosome appears to, at least partially, overrule the autoregulatory feedback loop. This suggests a model in which the activity of the minor spliceosome sets the expression level of *Srsf10*, which is then maintained through autoregulation.

**Figure 2—figure supplement 1.**
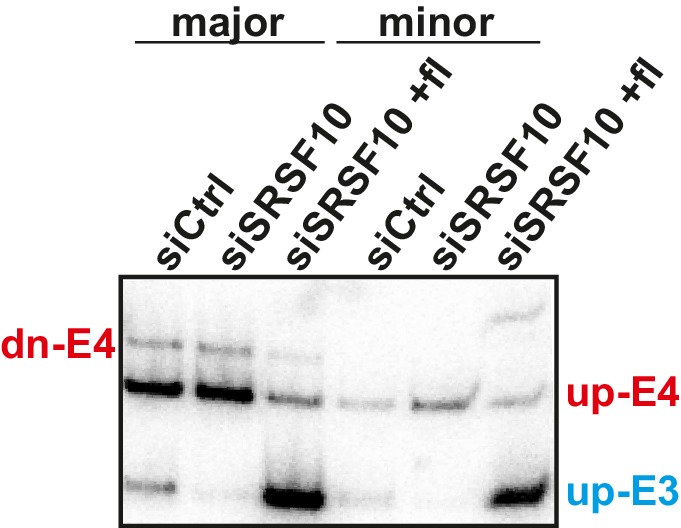
Competition of minor and major splice sites favors the downstream 5’ splice site A representative gel of only minor or major splice site containing minigenes. Minigene splicing of major and minor minigenes described and quantified in Figure 2A and B. Note that in the absence of competition between minor and major splice sites, we observe mainly splicing from the upstream 5’ splice site and the downstream 5’ splice site is rarely used.

### Minor spliceosome and SR protein expression correlate in a tissue-specific manner

**Figure 3.**
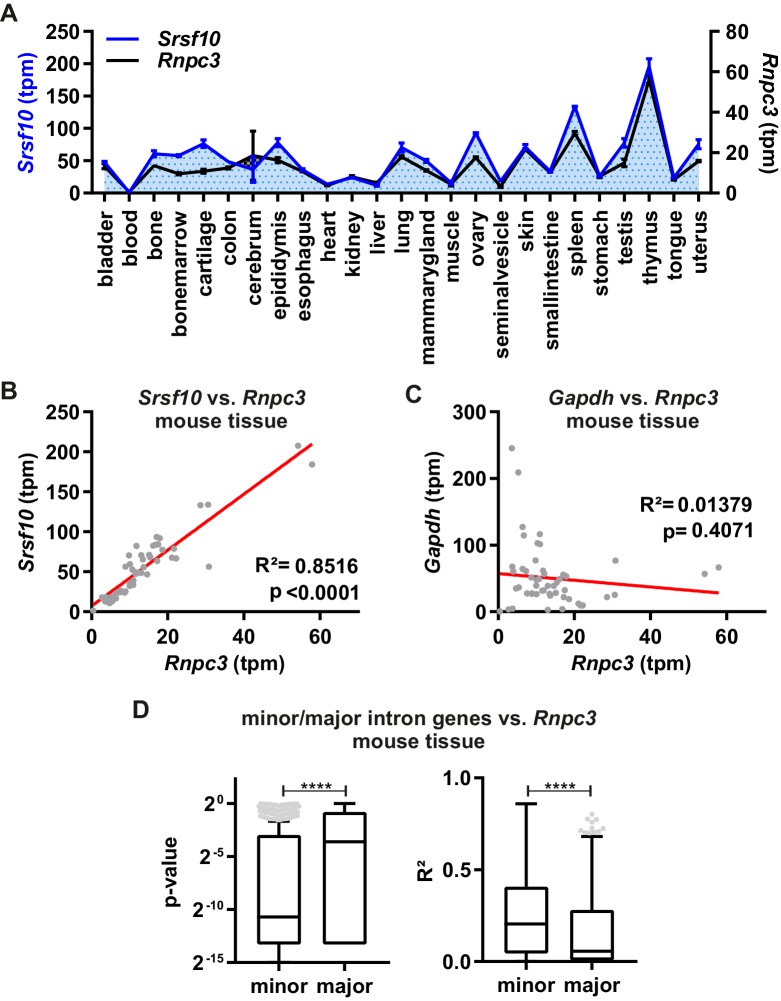
Minor spliceosome and Srsf10 expression correlate in a tissue-specific manner. (**A**) Relative GE levels of Srsf10 and Rnpc3 across 25 mouse tissues (x-axis). Transcripts per million (tpm) values were calculated using Whippet (Sterne-Weiler et al., 2018) (n ≥ 2, mean +/- SEM). (**B, C**) Linear regression fit for comparison of Srsf10 (**B**) and Gapdh (**C**) GE/tpm values with Rnpc3 across the 25 different mouse tissues. Goodness of fit is represented by R^2^ and p-values. (**D**) Calculated p-values (left) and R^2^ values (right) of a global correlation analysis of Rnpc3 with minor intron containing genes (n = 587) or randomly chosen expression matched genes, containing only major introns (n = 629). Statistical significance was determined by an unpaired t-test ****p<0.0001.

To investigate the relevance of this mechanism in vivo, we analyzed the correlation of *Srsf10* GE levels with expression of the minor spliceosome component *Rnpc3* across 25 different mouse tissues (Figure 3A). Calculated transcripts per million (tpm) values using Whippet Quant (Sterne-Weiler et al., 2018) revealed clear tissue-specific expression patterns for both *Srsf10* and *Rnpc3*. Both genes show the lowest GE in blood cells and the highest in thymus (Figure 3A). Notably, a linear regression fit revealed an almost perfect correlation of *Srsf10* and *Rnpc3* expression across the 25 investigated tissues (R²=0.85, p<0.0001, Figure 3B). Similarly, *SRSF10* and *RNPC3* GE levels correlate across 31 human tissues (R²=0.33, p=0.0006, Figure 3—figure supplement 1A). In contrast, the levels of the housekeeping gene *Gapdh*, which contains no minor intron, do not correlate with *Rnpc3* (R²=0.01, p=0.4071, Figure 3C). Similar correlation coefficients were obtained with gene expression values determined independently using Salmon (Patro et al., 2017; Figure 3—figure supplement 1B and C). Globally, minor intron-containing genes correlate much more strongly with *Rnpc3* levels than a randomly chosen group of expression level-matched genes containing only major introns (Figure 3D), indicating that *Rnpc3* levels represent an adequate indicator for minor spliceosome activity. Consistently, *Rnpc3* levels, and therefore *Srsf10* levels, also correlate with the expression levels of two other minor spliceosome components, namely *Snrnp25* and *Snrp48* (Figure 3—figure supplement 1D and E). These in vivo GE data are consistent with our model that minor spliceosome activity controls *Srsf10* levels. As an additional model system, we compared *Rnpc3* and *Srsf10* levels during neuronal differentiation of mouse embryonic stem cells (ES cells). GE levels correlate significantly (R²=0.34, p=0.0006, Figure 3—figure supplement 1F), while *Gapdh* levels do not correlate with *Rnpc3* (R²=0.02, p=0.4365, Figure 3—figure supplement 1G). Again, globally, minor intron-containing genes show a stronger correlation with *Rnpc3* levels than genes containing only major introns (Figure 3—figure supplement 1H). The *Srsf10*/*Rnpc3* correlation in ES cell differentiation is less pronounced – also with Salmon derived tpm values (Figure 3—figure supplement 1I) – indicating other factors influencing gene expression. Normalization of gene expression using DESeq2 (Love et al., 2014) strongly increases this correlation (Figure 3—figure supplement 1J), which is consistent with a direct role of the minor spliceosome in regulating *Srsf10* levels across different tissues and development stages. In summary, together with our minigene and knockdown results, these data indicate that the activity of the minor spliceosome controls productive vs unproductive *Srsf10* splicing and expression levels during development and in a tissue-specific manner.

**Figure 3—figure supplement 1.**
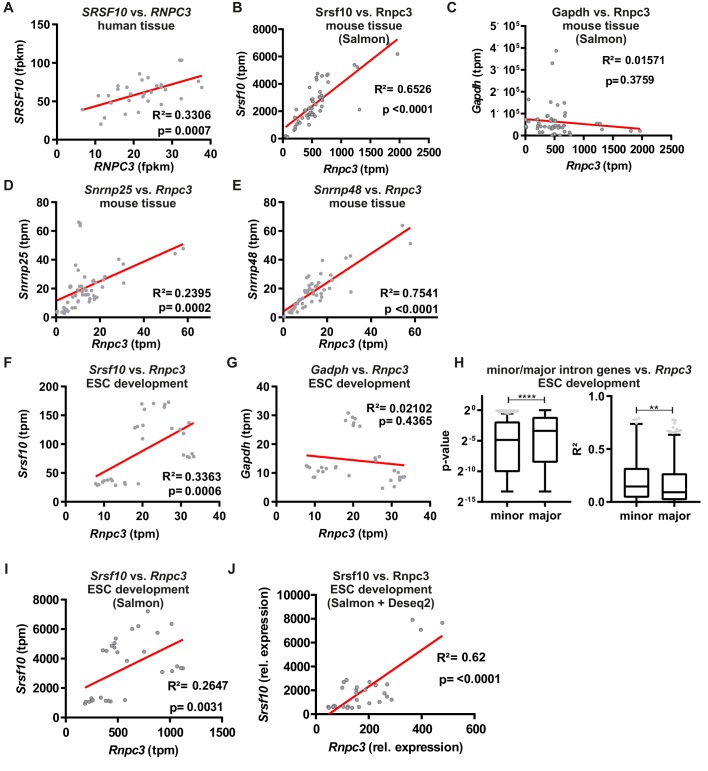
Minor spliceosome and Srsf10 expression correlate in a tissue-specific manner and during development. (**A**) Linear regression analysis of Srsf10 and Rnpc3 expression values (fpkm, fragments per kilobase of exon model per million reads mapped) across 31 human tissues (Uhlén et al., 2015). (**B, C**) Linear regression analysis of Srsf10 or Gapdh and Rnpc3 using Salmon derived tpm values. (**D, E**) Linear regression analysis of calculated tpm values for minor spliceosome components Snrnp25 (**D**) and Snrnp48 (**E**) against Rnpc3 in 25 mouse tissues. (**F, G**) Correlation analysis of calculated tpm values for Srsf10 (**F**) and Gapdh (**G**) against Rnpc3 in differentiating mouse ES cells (Hubbard et al., 2012). (**H**) Global correlation analysis of Rnpc3 to minor intron containing genes or randomly chosen expression matched genes containing only major introns. Calculated p-values (left) and R^2^ values (right) are shown. Statistical significance was determined by an unpaired t-test **p<0.01, ****p<0.0001. (**I, J**) Correlation analysis of calculated tpm values for Srsf10 against Rnpc3 in differentiating mouse ES cells using either Salmon-derived (**I**), or Salmon-derived and DESeq2-normalized (**J**) expression levels.

### SRSF10 and the minor spliceosome control tissue-specific and dynamic SR protein expression

**Figure 4.**
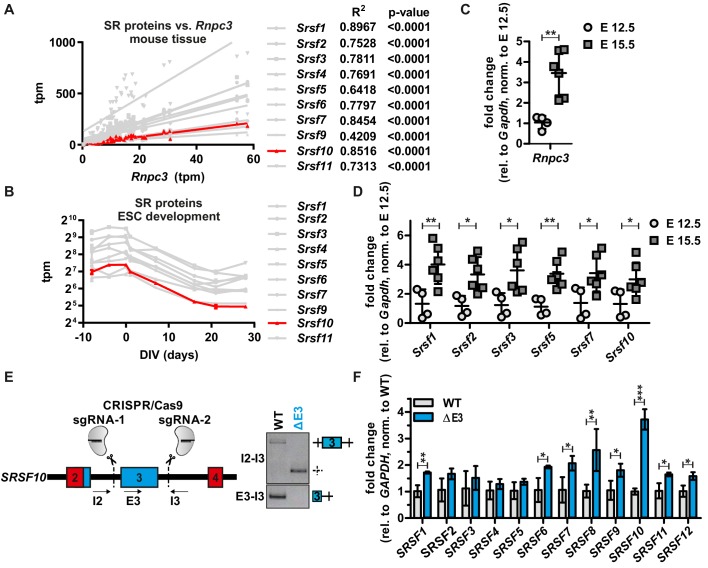
SRSF10 and the minor spliceosome control tissue-specific and dynamic SR protein expression. (**A**) Linear regression analysis of calculated tpm values for SR proteins against Rnpc3 in 25 different mouse tissues. R^2^ and p-values are shown on the right. See also Figure 4—figure supplement 1A with all SR proteins colored. Due to generally low expression levels Srsf12 was omitted. (**B**) Comparison of tpm values of SR proteins during mouse ES cell differentiation (n ≥ 3, mean ± SD). See also Figure 4—figure supplement 1B and C. (**C**) Rnpc3 GE levels in mouse cortex samples of the developmental stages E 12.5 and E 15.5 (relative to Gapdh, normalized to time point E 12.5) (n ≥ 4, mean ± SD). (**D**) SR protein expression in mouse cortex samples of the developmental stages E 12.5 and E 15.5. For Srsf10 only the functional up-E4 isoform was analyzed (relative to Gapdh, normalized to time point E 12.5) (n ≥ 4, mean ± SD). (**E**) Generation of clonal HEK293 cell lines lacking the regulatory Srsf10 exon 3. Left: Schematic of CRISPR/Cas9-mediated deletion of Srsf10 exon 3. Arrows indicate primer binding sites. Right: genotyping PCR on genomic DNA. (**F**) Relative SR protein expression in WT HEK293 cells or Srsf10ΔE3 cells. For Srsf10 only the functional up-E4 isoform was analyzed (relative to Gapdh, normalized to WT) (n = 3, mean +/- SD). Statistical significance was determined by an unpaired t-test *p<0.05, **p<0.01, ***p<0.001.

To investigate whether the minor spliceosome exclusively controls *Srsf10* expression, we next examined the expression levels of all other SR proteins in a tissue- and developmental stage-specific manner. Surprisingly, we found that the expression of all SR proteins correlates with the minor spliceosome, represented by *Rnpc3*, in the 25 investigated tissues (R²>0.42, p<0.0001, Figure 4A and Figure 4—figure supplement 1A). Additionally, we observed a highly similar expression pattern for all SR proteins during neuronal differentiation of mouse ES cells (Figure 4B and Figure 4—figure supplement 1B) and a significant correlation with *Rnpc3* (Figure 4—figure supplement 1C). To experimentally confirm these results in vivo, we isolated RNA from mouse cerebral cortices from embryonic days (E) 12.5 and 15.5. RT-qPCR *analysis* revealed a significant increase of *Rnpc3* GE from E 12.5 to E 15.5 and, in parallel, Srsf10 levels also increased (Figure 4C and D). This is consistent with our model that higher minor spliceosome activity (indicated by higher *Rnpc3* abundance) results in preferential usage of the *Srsf10* up-E4 minor splice site, which leads to an increase in protein coding *Srsf10* mRNA. Consistent with SR protein expression patterns from different tissues or stem cell development in RNA-Seq data (Figure 4A and B), all other tested SR proteins are also upregulated during the transition from E 12.5 to E 15.5 (Figure 4D), which, again, indicates a co-regulation of SR proteins, even though *Srsf10* is the only SR protein that contains a minor intron. As SR proteins are known to cross-regulate each other (Bradley et al., 2015), we hypothesized that a change in *Srsf10* levels could directly influence the levels of other SR proteins. To test this, we generated a CRISPR/Cas9-edited cell line lacking the non-productive *SRSF10* exon 3 (Figure 4E, left). Homozygous removal of exon 3 was confirmed by PCRs at the genomic level (Figure 4E, right) and, as expected, we observe increased *SRSF10* expression (Figure 4F, see Figure 4—figure supplement 1D for protein expression). Interestingly, we observe a stronger increase in the less active SRSF10-2 isoform (Figure 4—figure supplement 1D), indicating that AS of the last exon could be used to partially compensate for the loss of autoregulation via exon 3. Next, to examine whether manipulation of *SRSF10* autoregulation is sufficient to change the overall activity of SRSF10, we analyzed AS of SRSF10 target exons (Zhou et al., 2014; Wei et al., 2015). Consistent with increased *SRSF10* expression, the inclusion of alternative exons in *BCLAF1*, *PTBP2* and *ZFP207* is promoted in the CRISPR/Cas9-edited cells, and the opposite is observed by knockdown of *SRSF10* (Figure 4—figure supplement 1E). Notably, increased SRSF10 in our CRISPR/Cas9-edited cell line was sufficient for subtle upregulation of the mRNA levels of all other SR proteins (Figure 4F). In addition, reduced *SRSF10* expression upon *RNPC3* knockdown also correlates with slightly decreased expression of most other SR proteins (Figure 4—figure supplement 1F). In summary, these data suggest a model where minor spliceosome activity directly controls *SRSF10* levels, which is sufficient to change expression levels of the other SR proteins in a tissue- and differentiation state-specific manner (Figure 4—figure supplement 2). An exciting question that remains is how SRSF10 is able to coordinately regulate the abundance of the other SR proteins. Cross-regulatory mechanisms are known for many RBPs (Kumar and Lopez, 2005; Rossbach et al., 2009) and we therefore speculate that SRSF10 could regulate other SR proteins (and potentially other RBPs) by repressing the formation of NMD-targeted isoforms from their pre-mRNAs. This could be mediated either directly, by binding to the respective pre-mRNAs, or indirectly, through interactions with other SR proteins. Additionally, differences in SR protein abundance could be achieved by changing their activity. Higher mRNA abundance could be the consequence of reduced SR protein activity, resulting in reduced autoregulatory NMD exon inclusion and therefore higher mRNA expression levels (Ni et al., 2007). The activity of SR proteins is, amongst others, controlled by their phosphorylation level (Goldammer et al., 2018), and could be controlled by a direct or indirect effect of SRSF10 on the regulating kinases and phosphatases. While these mechanistic details remain to be investigated, the control of SR protein levels through the minor spliceosome and SRSF10 is of fundamental importance, as SR protein levels will have consequences for the splicing of most major introns. Our data thus reveal a mechanism through which the activity of the minor spliceosome controls major intron splicing in a tissue- and differentiation state-specific manner. This may also be relevant for diseases caused by minor spliceosome deficiencies (Jutzi et al., 2018; Verma et al., 2018), as misregulation of SR proteins and consequently, defects in major intron splicing (Cologne et al., 2019), may contribute to the observed phenotypes.

**Figure 4—figure supplement 1.**
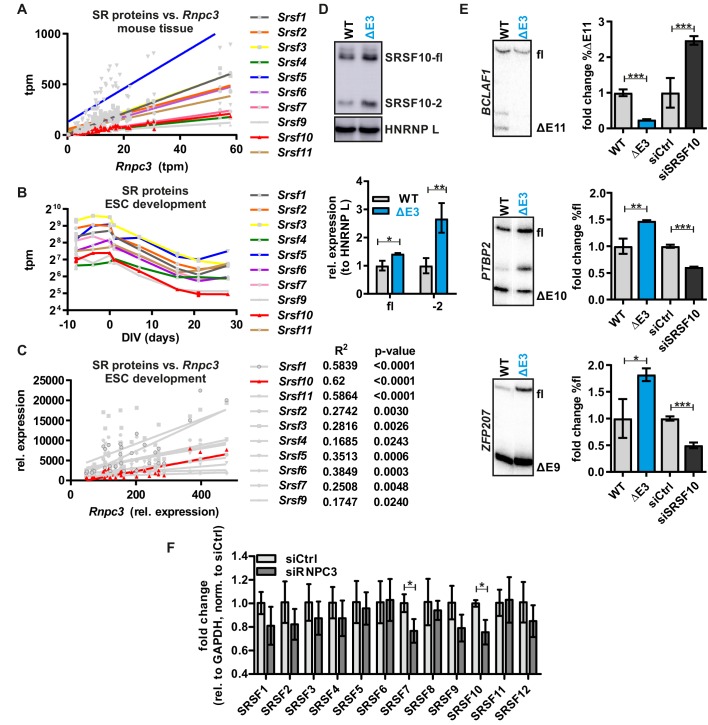
SRSF10 and the minor spliceosome control tissue specific and dynamic SR protein expression. (**A**) Linear regression analysis of calculated tpm values for the SR proteins against Rnpc3 in 25 different mouse tissues as shown in Figure 4A with all SR proteins colored. (**B**) Comparison of GE/tpm values of SR proteins during mouse ES cell differentiation as shown in Figure 4B with all SR proteins colored. (**C**) Linear regression analysis of SR proteins against Rnpc3 in neuronal differentiating mouse ES cells. R^2^ and p-values are shown on the right. Quantifications are based on Salmon and DESeq2 based expression levels. (**D**) SRSF10-fl and SRSF10-2 protein levels in CRISPR/Cas9-edited HEK293 cells (ΔE3) were analyzed by Western Blot and quantified relative to HNRNP L (n = 3, mean ± SD). (**E**) SRSF10 target gene splicing (top: Bclaf1, middle: Ptbp2, bottom: Zfp207) in WT and CRISPR/Cas9-edited cells (ΔE3) and after Srsf10 knockdown. Representative gels for radioactive RT-PCRs and quantifications of SRSF10 target gene splicing in ΔE3 cells and upon siRNA-mediated knockdown, relative to WT or siCtrl, respectively (n = 3, mean ± SD). (**F**) mRNA expression levels of SR proteins in HEK293 cells upon Rnpc3 knockdown. SR protein expression levels are normalized to siCtrl (n = 3, mean +/- SD). Student’s t test-derived p values *p<0.05, **p<0.01, ***p<0.001.

**Figure 4—figure supplement 2.**
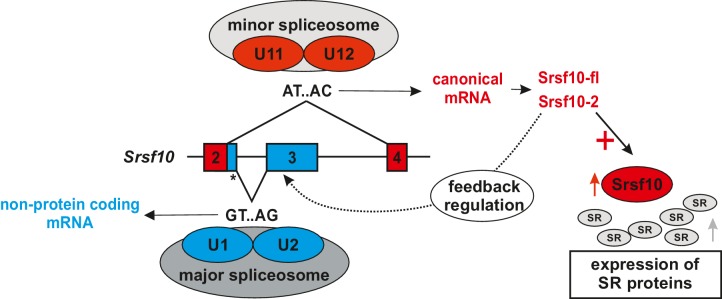
Model for minor spliceosome-mediated control of SR proteins AS of SRSF10 mirrors minor spliceosome activity. Reduced minor spliceosome activity results in increased formation of an unproductive SRSF10 variant through exon 3 inclusion via the major spliceosome. High minor spliceosome activity results in splicing to exon 4 and formation of protein-coding isoforms. SRSF10 regulates both its own expression level and the expression of other SR proteins.

## Materials and methods

**Key resources table:** 

Reagent type (species) or resource	Designation	Source or reference	Identifiers	Additional information
Gene (*Mus musculus*)	Srsf10	ncbi	GeneID:14105	Exons 2–4
Gene (*Homo sapiens*)	Gmfb	ncbi	GeneID:2764	Exon 4–5
Gene (*Homo sapiens*)	Srsf10	ncbi	GeneID:10772	
Cell line (*Homo sapiens*)	HeLa	ATCC	RRID:CVCL_0030	
Cell line (*Homo sapiens*)	HEK293	ATCC	RRID:CVCL_0045	
Cell line (*Homo sapiens*)	HEK293ΔE3	This paper		CRISPR/Cas9-mediated deletion of Srsf10 exon 3; see Figure 4 and Materials and methods Part
Biological sample (*Mus musculus*, NMRI strain)	Cortices	This paper		developmental stages E 12.5 and E 15.5; see Figure 4 and Materials and methods Part
Antibody	anti-FUSIP1 (T-18) (human, monoclonal)	Santa Cruz Biotechnology	RRID:AB_1123037	1:1000
Antibody	Anti-GFP (B-2)(monoclonal)	Santa Cruz Biotechnology	RRID:AB_627695	1:5000
Antibody	anti-Vinculin (H-300) (rabbit, polyclonal)	Santa Cruz Biotechnology	RRID:AB_2214507	1:1000
Antibody	anti-hnRNP L (4D11) (human, monoclonal)	Santa Cruz Biotechnology	RRID:AB_627736	1:10000
Recombinant DNA reagent	Mouse Srsf10 minigene	This paper		See Figure 1 + with Figure 1—figure supplements 1 and 2; and Materials and methods part
Recombinant DNA reagent	mouse Srsf10-fl-GFP	This paper		See Figure 1 + with Figure 1—figure supplements 1 and 2; and Materials and methods part
Recombinant DNA reagent	mouse Srsf10-2-GFP	This paper		See Figure 1 + with Figure 1—figure supplements 1 and 2; and Materials and methods part
Recombinant DNA reagent	mouse Srsf10-s-GFP	This paper		See Figure 1 + with Figure 1—figure supplements 1 and 2; and Materials and methods part
Recombinant DNA reagent	PX459 vector	Kindly provided by Stefan Mundlos	RRID:Addgene_62988	For sgRNA cloning and transfection
Recombinant DNA reagent	pEGFP-N3	Clontech		SRSF10 expression construct
Recombinant DNA reagent	pcDNA3.1(+)	Invitrogen	Catalog no: V79020	Minigene cloning
Sequence-based reagent	siRNA against human Srsf10 (siSrsf10)	This paper	GCGUGAAUUUGGUUAUdTdT	Knockdown of endogenous Srsf10
Sequence-based reagent	siRNA against human Rnpc3 (siRnpc3)	This paper	GAAAGAAGGUCGUAUGAAAdTdT	Knockdown of endogenous Rnpc3
Sequence-based reagent	Control siRNA (siCtrl)	This paper	UUUGUAAUCGUCGAUACCCdTdT	
Sequence-based reagent	sgRNA: SRSF10_E3_3fw	Benchling Tool	RRID:SCR_013955	Sequence: caccgctactttactcggtaagcca; CRISPR/Cas9-mediated deletion of Srsf10 exon 3
Sequence-based reagent	sgRNA: SRSF10_E3_3rv	Benchling Tool	RRID:SCR_013955	Sequence: aaactggcttaccgagtaaagtagc; CRISPR/ Cas9-mediated deletion of Srsf10 exon 3
Sequence-based reagent	sgRNA: SRSF10_E3_5fw	Benchling Tool	RRID:SCR_013955	Sequence: caccgtgagtttcagaagcatgaat; CRISPR/Cas9-mediated deletion of Srsf10 exon 3
Sequence-based reagent	sgRNA: SRSF10_E3_5rv	Benchling Tool	RRID:SCR_013955	sequence: aaacattcatgcttctgaaactcac; CRISPR/Cas9-mediated deletion of Srsf10 exon 3
Commercial assay or kit	PowerUp SYBR Green Mastermix	ThermoFisher Scientific	A25742	RT-qPCR
Chemical compound, drug	Roti-Fect	Carl Roth	Order no:P001.1	Plasmid vector transfection
Software, algorithm	GraphPad Prism 7.05	GraphPad	RRID:SCR_002798	Statistical analysis
Software, algorithm	ImageQuant TL	GE Life Sciences	RRID:SCR_014246	quantification
Software, algorithm	Whippet v0.11	Sterne-Weiler et al., 2018	RRID:SCR_018349	Tpm calculation
Software, algorithm	Salmon v1.2.0	Patro et al., 2017	RRID:SCR_017036	Tpm calculation
Software, algorithm	TxImport v1.14.0	Soneson et al., 2015	RRID:SCR_016752	Import of transcript counts to R for normalization wit DESeq
Software, algorithm	DESeq2 v1.26.0	Love et al., 2014	RRID:SCR_015687	Transcript counts normalization

### Tissue cell culture

HEK293 and HeLa cells were cultured in standard conditions. Transfections were done with Rotifect according to the manufacturer’s instructions. For siRNA sequences see Supplementary file 1. Minigenes were transfected 24 hr after knockdown and RNAs were isolated 48 hr later. For overexpression and rescue experiments, cells were transfected using 0.4 µg of minigenes and 0.4 µg expression vectors for GFP-tagged Srsf10-fl, Srsf10-2 or Srsf10-s (or GFP alone). 48 hr after transfection cells were harvested for protein and/or RNA preparation. We perform monthly test for mycoplasms using PCR. Cell have been tested negative in all tests during the experiments performed for the present study. The cells morphologically appear as expected for Hek293 and HeLa cells respectively. We have used these Hek293 cells in several RNA-Seq experiments and compared gene expression with published Hek293 datasets and found very good correlation.

### Preparation of embryonic mouse cortices for RNA extraction

Colonies of wild type mice of the NMRI strain were maintained in the animal facilities of Charité-Universitätsmedizin Berlin. Tissue collection was performed in compliance with German Animal Welfare Law and regulations imposed by the State Office for Health and Social Affairs Council in Berlin/Landesamt für Gesundheit und Soziales (LAGeSo).

Mice were bred for timed pregnancies, and the date of vaginal plug detection was considered embryonic day 0.5. Pregnancies were timed accordingly, and embryos prepared at the indicated embryonic days. Prior to the preparation of embryos, all tools were thoroughly cleaned with RNAse AWAY solution (Thermo Fisher, cat. no. 10328011). The uteri were dissected into DEPC-treated PBS, and the embryonic brains quickly transferred to a solution of 2M NaCl in PBS for RNA stabilization. Cortices were dissected and then snap frozen in liquid nitrogen.

### Molecular cloning

For cloning of the different Srsf10 overexpression constructs inserts were amplified from mouse cDNA and cloned into pEGFP-N3 (Clontech) using XhoI and BamHI restriction sites introduced through PCR primers. For minigene cloning, mouse genomic DNA was used as template to amplify *Srsf10* exons 2 to 4 and the product was cloned into pcDNA3.1(+). See Supplementary file 1 for cloning primer sequences. For mutational analysis *Srsf10* exons/introns were replaced by sequences from the human *GMFB* gene: exons 4 to 5, intron 4 is a minor intron. New inserts were amplified by 1-step or 2-step PCR and cloned into pcDNA3.1(+) using BamHI and XhoI or into the WT minigene using internal restrictions sites (BsrGI for exon 3 and XbaI for intron 3). In the I3 mutant 356 nt of Srsf10 exon 3 were maintained, the downstream sequence was replaced by the 5’ splice site of *GMFB* exon 4 (TCGatatcc…) and downstream sequence. For the E3 mutant sequences downstream of the BsrGI site (+16 in exon 3) were replaced by *GMFB* exon 4 to 5 sequence starting with (5’-CACCAGA…). For the ESE mutant nucleotids 17 to 60 of exon 3 were replaced by *GMFB* exon 4 sequence. For the minigene containing only minor splice sites we replaced the major splice sites of *Srsf10* intron 2 by minor splice sites of *GMFB* intron 4 (5’ splice site: 5’-TCGatatcc; 3’ splice site: 5’-ttctttaacttgagaaaaacCTT). In the minigene containing only major splice sites the upstream 5’ splice site of exon 2 and the 3’ splice site of exon 4 were replaced by major splice sites from *GMFB* intron 5 or 3, respectively (5’ splice site: 5’-TTGgtaagt; 3’ splice site: 5’-gcttttctctgtggtgccagGGC). All constructs were confirmed by sequencing.

### RT-PCR and RT-qPCR

RT-PCRs and RT-qPCRs were performed as previously described (Preußner et al., 2017). See Supplementary file 1 for primer sequences.

### Western blot

Western Blot analyses were done as previously described (Preußner et al., 2017). The following antibodies were used: αSRSF10/FUSIP1 (T-18, Santa Cruz), αGFP (B-2, Santa Cruz), αVINCULIN (H-300, Santa Cruz) and αHNRNPL (4D11, Santa Cruz) antibodies. Western blots were quantified using the ImageQuant TL software.

### RNA-Seq analysis

Transcripts per million (tpm) values were extracted from previously published RNA sequencing data using Whippet version 0.11 (Sterne-Weiler et al., 2018) and mouse reference genome mm10. Data for mouse tissues were obtained from WT control samples from SRA study DRP003641 (Tanikawa et al., 2017), data for neuronal differentiation of mouse ES cells from SRA study SRP017778 (Hubbard et al., 2012). Additionally, skin samples from SRP115206 were analyzed. For comparison, transcript abundances were additionally quantified using salmon version 1.2.0 (Patro et al., 2017). To obtain normalized expression counts, transcripts were imported to R using TxImport version 1.14.0 (Soneson et al., 2015) and count normalization was performed using DESeq2 version 1.26.0 (Love et al., 2014). DESeq2 based normalized expression counts dramatically increased the variance of replicate tissue samples (but not of ES cell differentiation), which is why we chose to analyze only the un-normalized values for the tissue comparisons. To investigate GE levels from different human tissues, fpkm values were directly used from Uhlén et al. (2015). Linear regression fits were performed in GraphPad Prism 7.05. A list of minor intron containing genes is based on Olthof et al. (2019). Genes with a comparable distribution of GE levels were randomly chosen from the remaining only major intron containing genes. The GE of each gene was compared to Rnpc3 levels across tissues or development stages. Unexpressed genes were excluded from the analysis. Downstream analyses were performed using standard Python 2 code and GraphPad Prism.

### Generation of CRISPR/Cas9-edited cell lines

For CRISPR/Cas9-mediated deletion of *SRSF10* exon 3 sgRNAs were designed using the Benchling tool (for sequences see Supplementary file 1) and cloned into the PX459 vector. Cells were transfected using Rotifect following the manufacturer's protocol. 48 hr after transfection, transfected cells were selected with 1 μg/ml puromycin and clonal cell lines were isolated by dilution (Ran et al., 2013). Genomic DNA from clonally expanded lines was extracted and analyzed by PCR.

## Data Availability

All data are previously published and publicly available. The following previously published datasets were used: Human Genome Center, The University of Tokyo2017p53 mouse multi-tissue transcriptome analysisNCBI Sequence Read ArchiveDRP003641 USAMRICD2012Deep transcriptional profiling of longitudinal changes during neurogenesis and network maturation in vivoNCBI Sequence Read ArchiveSRP017778

## Additional files

Supplementary file 1. Oligonucleotide sequences of primers, siRNAs, and guide RNAs.

Transparent reporting form
